# Umbilical Cord Procalcitonin to Detect Early-Onset Sepsis in Newborns: A Promising Biomarker

**DOI:** 10.3389/fped.2021.779663

**Published:** 2021-12-10

**Authors:** O. R. E. Dongen, L. M. van Leeuwen, P. K. de Groot, K. Vollebregt, I. Schiering, B. A. Wevers, S. M. Euser, M. A. van Houten

**Affiliations:** ^1^Department of Paediatrics, Spaarne Hospital, Haarlem, Netherlands; ^2^Department of Paediatrics, Willem Alexander Children Hospital, Leiden University Medical Center, Leiden, Netherlands; ^3^Department of Gynaecology, Spaarne Hospital, Haarlem, Netherlands; ^4^Department of Clinical Chemistry, Atalmedial Medical Diagnostic Centers, Haarlem, Netherlands; ^5^Laboratory for Medical Microbiology, Regional Public Health Laboratory Kennemerland, Haarlem, Netherlands

**Keywords:** procalcitonin, early-onset sepsis, neonatal infection, umbilical cord blood, antibiotic stewardship

## Abstract

**Background:** Up to 7% of neonates born in high-income countries receive antibiotics for suspected early-onset sepsis (EOS). Culture-proven neonatal sepsis has a prevalence of 0.2%, suggesting considerable overtreatment. We studied the diagnostic accuracy of umbilical cord blood and infant blood procalcitonin (PCT) in diagnosing EOS to improve antibiotic stewardship.

**Methods:** Umbilical cord blood PCT was tested in newborns ≥ 32 weeks of gestation. Groups were defined as following: A) culture-proven or probable EOS (*n* = 25); B) Possible EOS, based on risk factors for which antibiotics were administered for <72 h (*n* = 49); C) Risk factor(s) for EOS without need for antibiotic treatment (*n* = 181); D) Healthy controls (*n* = 74). Additionally, venous or capillary blood PCT and C-reactive protein (CRP) were tested if blood drawing was necessary for standard care.

**Results:** Between June 2019 and March 2021, 329 newborns were included. Umbilical cord blood PCT was significantly higher in group A than in group C and D. No difference between venous or arterial samples was found. Sensitivity and specificity for cord blood procalcitonin were 83 and 62%, respectively (cut-off 0.1 ng/mL). Antepartum maternal antibiotic administration was associated with decreased PCT levels in both cord blood and infant blood directly postpartum in all groups combined.

**Conclusion:** Umbilical cord blood PCT levels are increased in newborns ≥32 weeks with a proven or probable EOS and low in newborns with risk factors for infection, but PCT seems not a reliable marker after maternal antibiotic treatment. PCT could be useful to distinguish infected from healthy newborns with or without EOS risk factors.

## Introduction

Bacterial early-onset sepsis (EOS) is a systemic infection presenting within the first week after birth. It is a severe condition that can be lethal within hours, making rapid diagnosis essential (1, 2). EOS is defined as transplacental infection or ascending infection from the maternal genital tract with *S. agalactiae* (GBS) and *E. coli* as the most common causative agents (1). In high-income countries, the incidence of a blood-culture proven EOS is relatively low, with 0.5–2 per 1,000 newborns, accounting for 85–340 patients per year in the Netherlands (3, 4). However, an estimated 5–8% of all newborns (8.500–13.600 patients per year in the Netherlands) are treated with antibiotics in the first days after birth, and adherence to the EOS guidelines is low (5, 6). Initial presentation can be subtle, and symptoms mimic physiological phenomena associated with birth. This challenges the clinician to identify the newborn with an infection and drives the massive use of antibiotics for suspected EOS (4). The problem of increasing resistance due to the overuse of antibiotics is well-recognized. In addition, evidence is growing that antibiotic therapy early in life may change the individual's microbiome, with possible consequences for the developmental origins of future health and disease (7, 8).

The current golden standard for diagnosing EOS is a positive blood culture, but a bacterial cause is often not identified (3). Guidelines are in place to further aid EOS diagnosis and are based on clinical symptoms, maternal risk factors or maternal GBS screening (9, 10). Additionally, an interactive and easy to use tool, the EOS calculator, has been developed to help clinicians decide whether to start with antibiotics or not (11). With the implementation of these screening instruments, antibiotic use has been reduced, but EOS incidence has not declined over time, and detection has not improved (12–15).

Several biomarkers are being studied as promising candidates to further improve EOS recognition, such as the acute phase protein procalcitonin (PCT). PCT has proven its use as an infection parameter in preterm and full-term newborns and children (16–18). Advantages include a relatively short half time with fast peak levels and its minor increase in response to other factors than bacterial infection (Eschborn and Weitkamp, 2019). Moreover, PCT-guided decision making can be used safely to reduce the duration of antibiotic treatment (19, 20). A disadvantage of PCT is its physiological increase within 48–72 h postpartum, corresponding with the presentation and diagnosis of EOS. Umbilical cord sampling precedes this physiological peak and has shown to be a reliable method to aid decision making in an early stage (21). Recently, it has been shown that an umbilical cord PCT-based algorithm has the potential to decrease the number of preterm newborns that receive antibiotic therapy (22). Until now, no study has focused on the potential difference between venous umbilical cord blood, which originates from the mother or placenta, and arterial cord blood, originating from the newborn.

Although research has focused on umbilical cord blood PCT as a promising candidate in EOS diagnosis, studies have been heterogeneous in study design and EOS definition. The main objective of this study is to investigate if PCT is an accurate diagnostic biomarker in late preterm and full-term children to distinguish infected from healthy newborns with or without risk factors. In addition, we will evaluate the effect of maternal antibiotics on umbilical cord blood PCT and the potential difference between PCT in arterial vs. venous cord blood samples.

## Materials and Methods

### Study Design

This is a prospective, single-center, case-control study with participants enrolled in the Spaarne Hospital, Haarlem. Spaarne Hospital is a tertiary teaching hospital covering an urbanized area in the western part of the Netherlands, with 2640 newborns per year (excluding non-medical deliveries). The study period was between June 15, 2019, and March 28, 2021.

All parents provided informed consent before any data was collected. The Medical Ethical Committee of the Amsterdam UMC approved this study on March 18th, 2019 (ID: NL67266.029.18). The data was anonymized after collection and analyzed under code.

### Participants

All newborns born after a gestational age of ≥32 weeks were eligible for inclusion. Newborns were included directly after birth when risk factors for infection were present. Additional newborns without risk factors but with clinical signs of infection were included within the first week after birth. The risk factors and clinical signs used in this study are described in the Dutch national guidelines (Supplementary Table 1). Newborns with (suspected) chromosomal abnormalities or serious congenital disorders, as well as newborns previously admitted to another hospital, were excluded from this study.

Newborns were categorized as follows. Group A consisted of newborns with proven and probable EOS and patients in this group were defined as cases. Proven EOS was defined as a positive blood culture for *E. coli* or GBS. Probable EOS was based on at least two maternal and/or clinical risk factors at presentation, prolonged symptoms of infection and a serial CRP >10 mg/l [Supplementary Table 2, (3)]. Group B consisted of newborns with a possible infection, defined as one or more maternal and/or clinical risk factors for which antibiotic treatment was started. If the clinical condition was satisfactory within the following days and serial CRP testing showed a low value (<10 mg/l) the possibility of EOS was ruled out and antibiotic treatment was discontinued within 72 hours. Group C consisted of newborns with one or more maternal and/or clinical risk factors yet who did not receive antibiotic treatment because of a clinically low EOS suspicion. Both groups B and C are considered controls. Cases and controls were included in a 1:5 ratio, with 15 cases and 75 controls. Finally, 90 newborns without any risk factors or clinical signs for infection were included as an extra control group (Group D) to study baseline PCT levels in the current cohort.

### Study Procedure

Venous and arterial umbilical cord blood samples were collected directly after the placenta was born and after umbilical cord clamping. This was done for all newborns within the defined inclusion criteria. When a newborn was suspected of infection, blood samples for culture and CRP were drawn, and antibiotic treatment was started, according to our local protocols. CRP follow-up testing was performed 24-48 hours after initiation of antibiotic therapy. These tests are part of standard care in cases of EOS suspicion. To avoid the need for multiple venipunctures, PCT testing was only performed when CRP samples were needed according to the protocol and tests were performed in the same sample. PCT analysis was done batch-wise at a later timepoint and was not conveyed to the pediatrician in charge to avoid treatment adaptation based on PCT results.

Clinical observation and additional laboratory result data were reported by the pediatrician and recorded in the Electronic Patient Record (EPR). This data was collected in the study database by the researcher and included: body temperature, blood pressure, capillary refill, respiratory rate, heart rate, oxygen saturation, neurological state, urinary output, the pediatricians' notes regarding the estimated severity of EOS, blood culture results, full blood count with differential, CRP, and glucose.

Antibiotic treatment was started according to the national guideline and the local protocol. During the study, there was an adaptation of the local guideline for the treatment of EOS. The initial regimen of amoxicillin and cefotaxime was replaced by one of benzylpenicillin with gentamycin (23).

### Sample Processing and PCT Analysis

For the collection of umbilical cord blood samples, heparin gel-coated tubes (Beckton Dickson heparin Microtainer) were used. The collected samples were centrifuged to obtain the plasma fraction and stored at−80 °C for later PCT analysis. PCT was then analyzed from these frozen plasma samples. The BRAHMS PCT chemiluminescent microparticle immunoassay (CMIA) (0.02-100 ng/ml measuring range) and the Thermo Scientific BRAHMS PCT sensitive KRYPTOR immunoassay (0.02 - 50 ng/ml measuring range) were used for measuring PCT.

### Outcome

The primary outcomes of this study were the sensitivity, specificity, and the positive and negative predictive values of PCT in umbilical cord blood, as well as in venous or capillary follow-up samples postpartum.

The secondary outcome of this study was the influence of maternal antibiotics on PCT measured in the umbilical cord, the difference between PCT in arterial and venous cord blood samples, and the level of CRP measured postpartum in the newborn with a (suspected) infection.

### Statistical Analysis

Descriptive statistical analysis was done to identify the incidence of EOS in our study group; baseline clinical characteristics were evaluated. Data was checked for normality of distribution using both visual analysis and Shapiro-Wilk's testing. A mean and standard deviation for normally distributed data and median with interquartile range for non-normally distributed data was calculated for demographic parameters, clinical parameters, and results of laboratory tests. Additionally, CRP and PCT plots were made of individual newborns to demonstrate biomarker dynamics. Group comparisons of independent samples were made using a Wilcoxon signed-rank test with Bonferroni correction for multiple testing. Sensitivity and specificity were calculated for PCT using cross-tabulation, and ROC graphs were performed for PCT in umbilical cord blood samples and PCT and CRP directly postpartum. The Youden index was used to determine the optimal cut-off value by ROC analysis.

All statistical analysis was done using R-Studio version 1.2.1335 (R-Studio, Inc.). In statistical analysis, *p-*values < 0.05 were considered statistically significant.

## Results

### Study Population and Risk Factors

**Figure 1:**
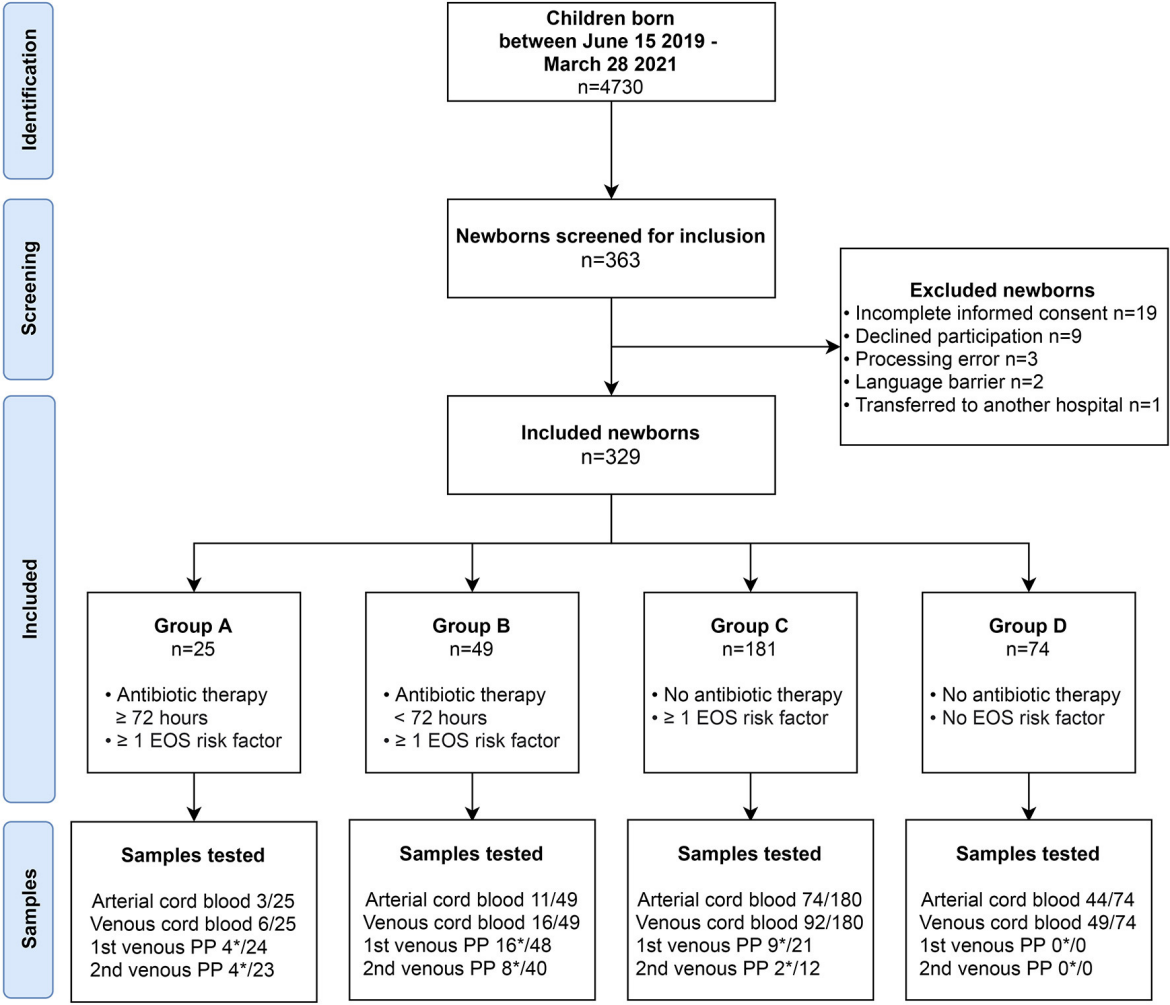
Study design. Flow diagram demonstrating identification, screening and inclusion of participants. Group A: Newborns with proven or probable EOS and >72 h antibiotic treatment. Group B: Newborns with possible EOS, but antibiotics were discontinued <72 h. Group C: newborns with one risk factor for EOS that were not treated with antibiotics. Group D: Healthy controls. Asterisk (*) indicates the number of successfully executed PCT tests of the total available blood samples.

Between June 15, 2019, and March 28, 2021, a calculated number of 4730 newborns were born at ≥32 weeks gestational age in the Spaarne Gasthuis Hospital, of whom 329 newborns were included in the study (Figure 1). Twenty-five newborns presented with a proven or probable EOS and received antibiotic treatment for >72 hours (Table 1, Group A). In two newborns blood culture-proven bacteremia with *Streptococcus agalactiae* (GBS) was identified. In three cases, pathogens considered to be contaminants were detected (*Enterococcus faecalis, Staphylococcus epidermidis, and Bacillus weihenstephanesis)*, of which two patients were included in group A based on clinical presentation and one patient was included in group B. Group B consisted of 49 newborns with a possible EOS that received antibiotics for a maximum of 72 hours. Antibiotics were discontinued when the possibility of EOS was ruled out. Control groups C and D consisted of 181 and respectively 74 newborns. In these groups no antibiotics were prescribed.

**Table 1:** Baseline characteristics.

	**Group A Proven / probable EOS**	**Group B Possible EOS**	**Group C No EOS**	**Group D Baseline**	***p*-value Comparing Groups A, B and C**
**Demographics**					
	25	49	181	74	
Female Gender [n (%)]	12 (52.2)	15 (33.3)	94 (53.4)	36 (52.9)	0.054
Gestational Age, weeks [mean (range)]	39.38 (39.57–41.00)	37.79 (35.00–40.64)	38.61 (37.00–40.43)	39.41 (38.57–40.43)	**0.023**
Birth Weight, grams [mean (SD)]	3417.36 (789.99)	3052.81 (870.03)	3297.58 (702.70)	3443.39 (583.93)	0.074
Delivery method [n (%)]					**0.010**
Spontaneous vaginal	15 (62.5)	24 (51.1)	134 (75.7)	55 (78.6)	
Vacuum or forceps	2 (8.3)	12 (25.5)	23 (13.0)	11 (15.7)	
Elective cesarean section	2 (8.3)	1 (2.1)	4 (2.3)	0 (0.0)	
Secondary cesarean section	5 (20.8)	10 (21.3)	16 (9.0)	4 (5.7)	
Venous cord blood pH [mean (SD)]	7.24 (0.12)	7.24 (0.09)	7.22 (0.41)	7.24 (0.09)	0.961
Arterial cord blood pH [mean (SD)]	7.18 (0.11)	7.17 (0.08)	7.14 (0.62)	7.18 (0.07)	0.929
Apgar					
1 min [mean (SD)]	7.33 (2.16)	7.87 (2.00)	8.61 (1.25)	8.74 (0.83)	**<0.001**
5 min [mean (SD)]	8.58 (1.69)	9.00 (1.57)	9.60 (0.94)	9.77 (0.47)	**<0.001**
10 min [mean (SD)]	8.58 (1.24)	9.09 (1.53)	9.71 (0.83)	10.00 (0.00)	**0.006**
Maternal antibiotics (%)	5 (20.8)	26 (55.3)	99 (55.6)	5 (6.8)	**0.005**
**Reported maternal risk factors [n(%)]**					
GBS positive mother	3 (12.0)	8 (16.3)	9 (5.0)		**0.023**
Maternal AB treatment <24 h postpartum	0 (0.0)	4 (8.2)	0 (0.0)		**<0.001**
Invasive GBS infection in a previous child	1 (4.0)	0 (0.0)	5 (2.8)		0.448
Maternal GBS colonization, bacteriuria or infection in the current pregnancy	0 (0.0)	3 (6.1)	6 (3.3)		0.385
Cystitis in current pregnancy	0 (0.0)	1 (2.0)	0 (0.0)		0.121
PROM >24 h in a term pregnancy	4 (16.0)	10 (20.4)	94 (51.9)		**<0.001**
Suspected or confirmed PROM ≥18 h in a preterm birth	1 (4.0)	10 (20.4)	18 (9.9)		0.058
Spontaneous preterm birth (gestational age <37 weeks)	3 (12.0)	13 (26.5)	33 (18.2)		0.267
Intrapartum fever >38°C, or confirmed/suspected chorioamnionitis	10 (40.0)	21 (42.9)	32 (17.8)		**<0.001**
**Reported neonatal risk factors [n (%)]**					
Respiratory distress >4 h pp	3 (12.0)	7 (14.3)	4 (2.2)		**0.001**
Signs of shock	1 (4.0)	1 (2.0)	0 (0.0)		0.056
Behavioral change	0 (0.0)	6 (12.2)	4 (2.2)		**0.003**
Trouble with feeding (refusal of feeding, gastric retention, vomiting, abdominal distension)	2 (8.0)	0 (0.0)	1 (0.6)		**0.004**
Apnea	0 (0.0)	1 (2.0)	0 (0.0)		0.121
Bradycardia	0 (0.0)	1 (2.0)	0 (0.0)		0.121
Signs of respiratory distress	17 (68.0)	14 (28.6)	16 (8.9)		**<0.001**
Hypoxia (%)	2 (8.0)	3 (6.1)	5 (2.8)		0.304
The necessity for mechanical ventilation in a premature newborn	0 (0.0)	2 (4.1)	1 (0.6)		0.107
Fever (>38°C) of hypothermia (<36°C) not explained by environmental factors	4 (16.0)	7 (14.3)	1 (0.6)		**<0.001**
Local signs of infection (e.g., on the skin or eyes)	3 (12.0)	1 (2.0)	1 (0.6)		0.001
**Other [mean (SD)]**					
Sum of maternal risk factors	0.88 (0.93)	1.43 (1.00)	1.09 (0.49)		**0.001**
Sum of neonatal risk factors	1.28 (1.14)	0.88 (1.01)	0.18 (0.50)		**<0.001**
Sum of all risk factors combined	2.16 (1.28)	2.31 (1.19)	1.27 (0.59)		**<0.001**
Duration of PROM (h)	22.48 (38.16)	56.44 (121.59)	55.44 (158.53)	10.10 (8.10)	0.609
Duration of antibiotic treatment (h)	165 (61.15)	42.77 (17.38)			**<0.001**
Highest maternal temp during labor in °C	37.83 (0.92)	37.86 (0.94)	37.47 (0.63)	37.15 (0.42)	**0.003**

*Data are n (%), mean (SD) or mean (range). p-values are derived from comparing groups A, B and C; excluding baseline group D*.

*PROM, premature rupture of membranes. Behavioral change: a silent newborn or hypotonia. Signs of respiratory distress, tachypnoea, groaning, retractions or nasal flaring. Hypoxia: central cyanosis or low oxygen saturation. Local signs of infection, skin or eye changes related to infection*.

As expected, based on group definition, the mean number of documented risk factors was significantly higher in group A and B in comparison with group C. Even though the amount of risk factors in group A and B was similar, a difference was found when comparing type of risk factor at presentation. Newborns of group A presented more often with neonatal clinical signs than group B, for example with signs of respiratory distress (Table 1). In contrast, group B predominantly consisted of mothers admitted to the hospital due to maternal risk factors such as premature birth (GA <37 weeks) and premature rupture of membranes. Intrapartum fever >38°C was the most prevalent maternal risk factor described in both groups (Group A: 40%, Group B: 42.9%, Group C: 18.8%) which resulted in prescription of antibiotics.

The duration of antibiotic treatment was continued for a mean of 6.9 days in group A and 1.8 days in group B (*p* < 0.001). Of all neonates treated with antibiotics; 59 (63,5%) s received amoxicillin with cefotaxime, and 34 (36.5%) newborns received benzylpenicillin with gentamycin.

### Umbilical Cord Procalcitonin

To study the usability of PCT as an early diagnostic marker for EOS, PCT levels were tested in umbilical cord samples for all newborns. When comparing PCT levels in arterial vs. venous blood samples, no significant differences were found (results not shown). This indicates that sample type did not affect PCT levels in the current cohort. Since no differences were found between arterial and venous PCT, one sample per newborn was used. When both samples were available, the venous sample was selected.

**Figure 2:**
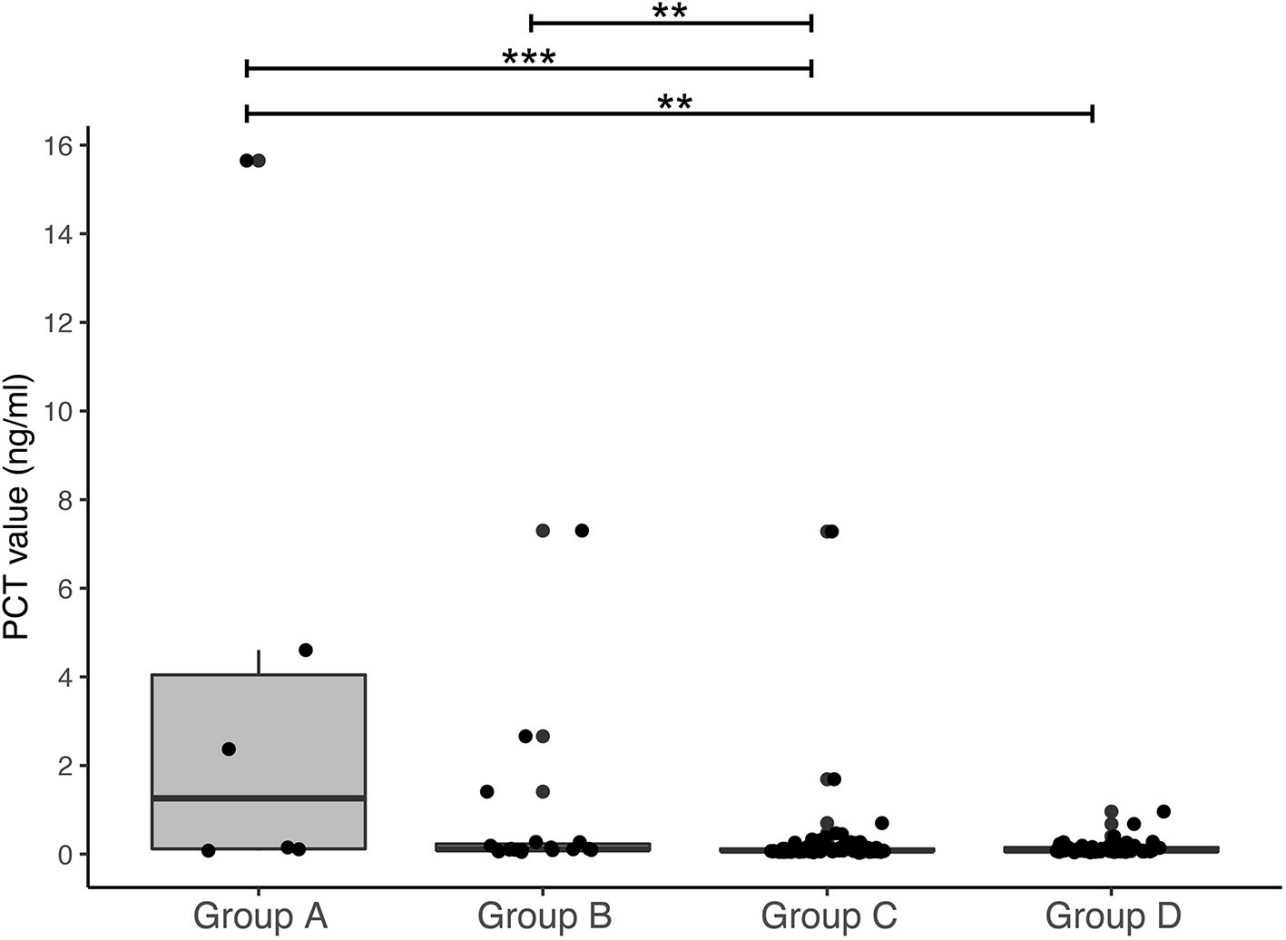
Procalcitonin in umbilical cord blood. Boxplot representing cord blood levels of procalcitonin (arterial and venous samples combined per group). Plots show dots per individual PCT level projected on top of boxplot with median and interquartile range. Counts per group: Group A: *n* = 9 (arterial *n* = 3, venous *n* = 6); Group B: *n* = 27 (arterial *n* = 11, venous *n* = 16); Group C: *n* = 166 (arterial *n* = 74, venous *n* = 92); Group D: *n* = 93 (arterial *n* = 44, venous *n* = 49). **p < 0.05, ***p < 0.01.

Umbilical cord blood PCT levels were significantly higher in samples of group A compared to groups C and D (Figure 2, Mann-Whitney U, *p* = 0.009 and *p* = 0.02 respectively). Cord blood PCT in group B samples were significantly higher than in samples of group C (*p* = 0.02). No significant differences were found when comparing PCT in groups B vs. D and groups C vs. D (*p* = 0.06 and *p* = 0.86 respectively).

### Sequential CRP and PCT Testing

**Figure 3:**
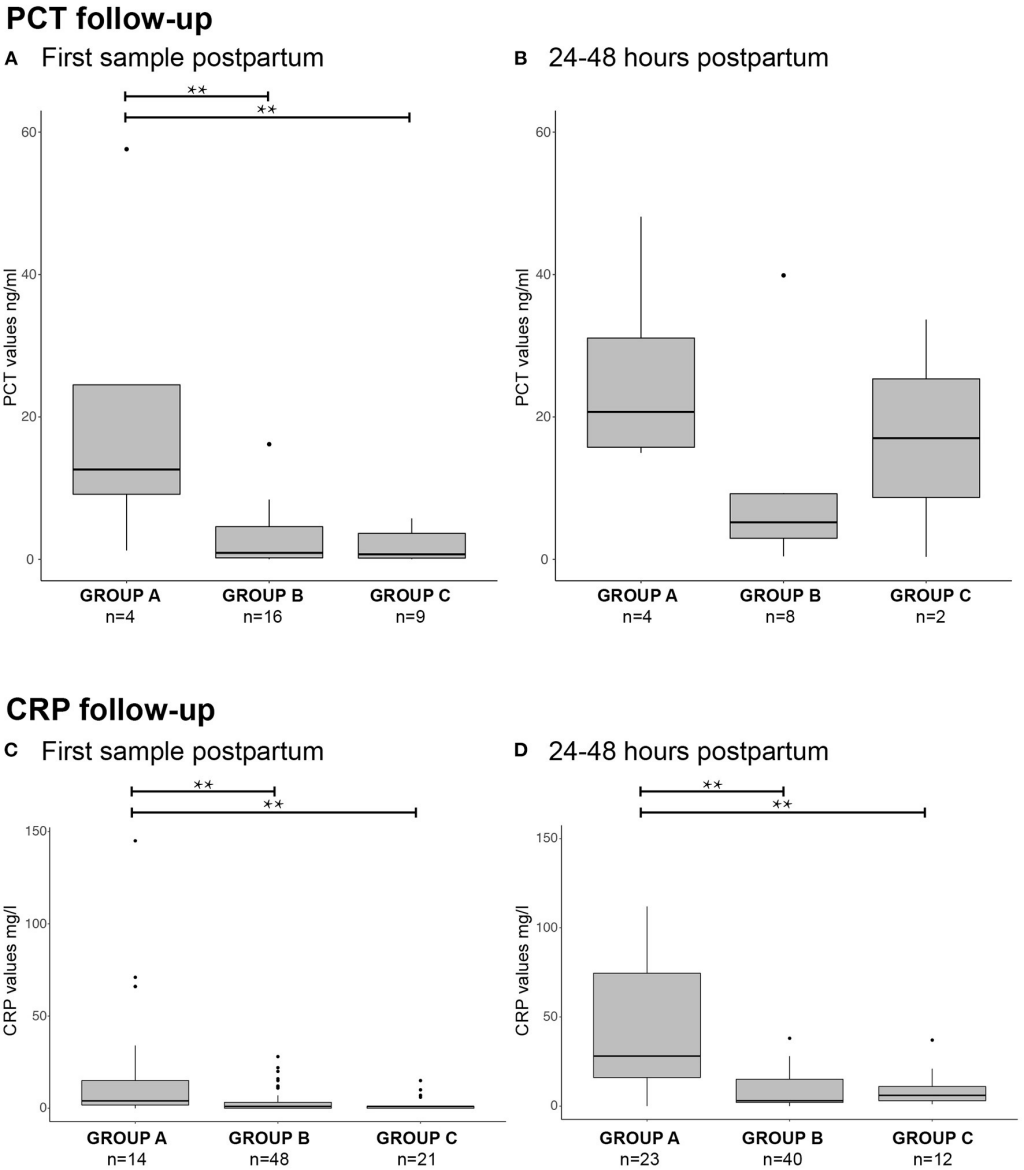
PCT and CRP postpartum. Boxplots with outliers (small dots) showing PCT and CRP values directly postpartum [**(A)**: PCT, **(C)**: CRP] and 24–48 h postpartum [**(B)**: PCT, **(D)**: CRP] for groups A, B and C. Samples per group are displayed as *n* = x at the corresponding boxplot. **p < 0.05.

To determine the accuracy of PCT and CRP in blood samples postpartum, these levels were compared between groups A, B, and C (Figure 3). Newborns in group A displayed an elevated PCT directly after birth (median PCT: 12.63 ng/ml, IQR 1.25 – 24.51), which was significantly higher than presented in both groups B and C (group B: median PCT 0.91 ng/ml, IQR 0.21 – 4.6. Group C: median PCT 0.72 ng/ml, IQR 0.17 – 3.66. Mann-Whitney U group A vs. B = 0.029, A vs. C = 0.05, A vs. B-C combined *p* < 0.01, Figure 3A). Newborns in Group C (*n* = 9) were tested for CRP and PCT due to a suspicion of EOS. However, they were not treated with antibiotics due to a low CRP with a normal leukocyte count and fast clinical improvement. Follow-up measurements after a median of 32 hours (IQR 27 – 41) showed a median PCT of 20.7 ng/ml (IQR 14.96 – 31.09) in group A. At this time point, no significant difference was found between groups (Figure 3B, *p* = 0.125). CRP values were significantly higher in group A than groups B and C for samples taken directly postpartum and at 24–48 hours follow-up (Figures 3C,D; *p* < 0.05). In summary, our data show that newborns in group A with a high suspicion of EOS have an increased PCT and CRP in umbilical cord blood and in blood samples directly postpartum.

### Antepartum Antibiotic Treatment

**Figure 4:**
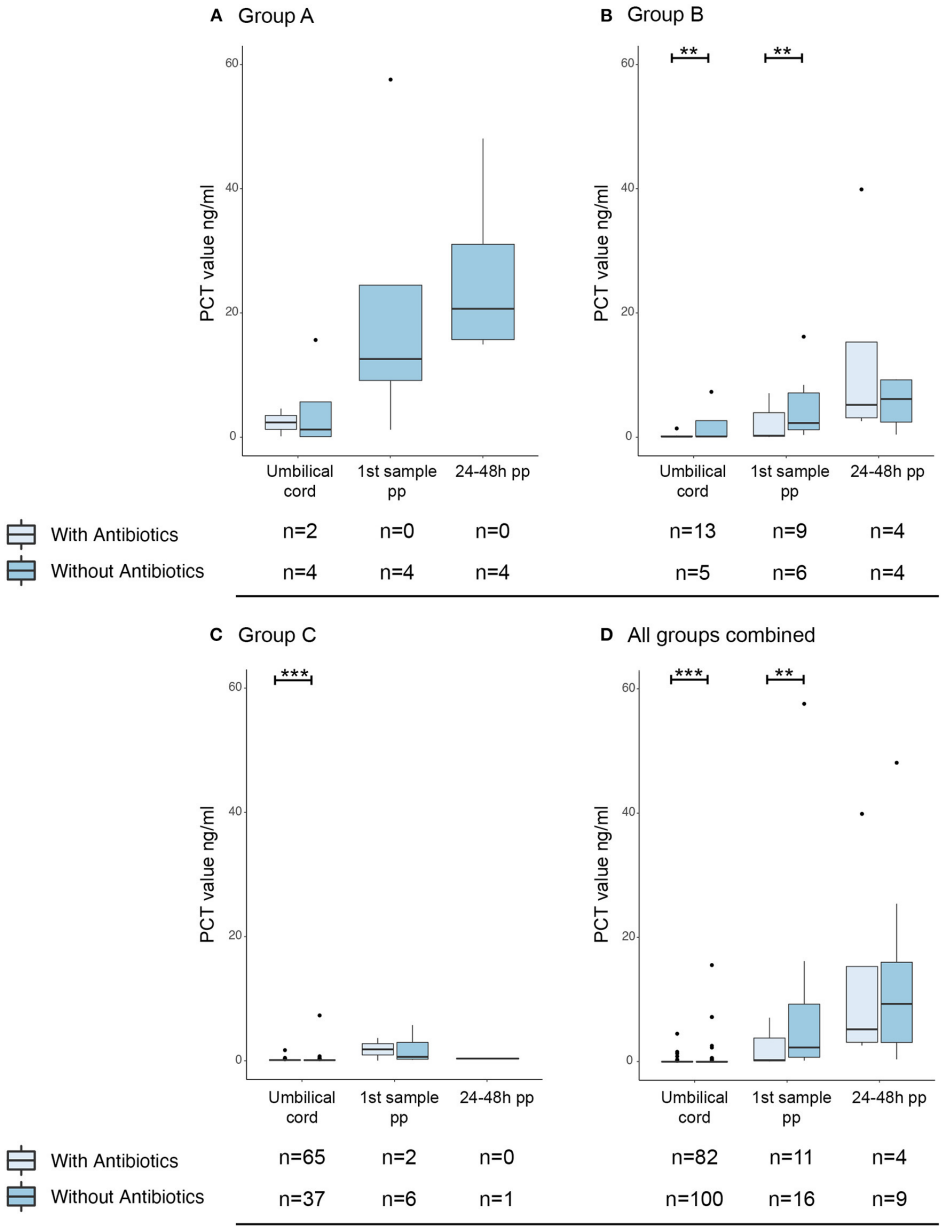
Effect of maternal antibiotic treatment antepartum on PCT levels. Boxplots with outliers (dots) of PCT in umbilical cord blood, directly postpartum and 24–48 h postpartum. A separation was made for group A **(A)**, group B **(B)**, group C **(C)** and all groups combined **(D)**. For umbilical cord blood samples, arterial and venous PCT levels were combined. Per sample moment results are stratified by whether the mother was given prophylactic antibiotic treatment (light blue) or not (darker blue). Numbers per group are presented under the corresponding boxplot. pp: postpartum. **p < 0.05; ***p < 0.01.

A significant proportion of mothers received antibiotic treatment, due to GBS positivity or suspected infection during delivery (Table 1). Interestingly, maternal antibiotic treatment was administered significantly less often in group A (20.8% of cases) compared to group B and C (55.3% and 55.6% resp. of all cases). To examine the effect of antepartum antibiotics, PCT levels in umbilical cord and infant blood were compared for these groups. Newborns from mothers with antepartum antibiotic treatment had a significantly lower umbilical cord blood PCT than the newborns whose mothers had not received antibiotic treatment (Figure 4B, *p* = 0.002; Figures 4C,D, *p* = 0.009). Similarly, PCT in postpartum blood was significantly lower in newborns of group B and all groups combined when antibiotics were administered antepartum (*p* = 0.004 and *p* = 0.001 respectively).

### Sensitivity, Specificity and ROC

**Figure 5:**
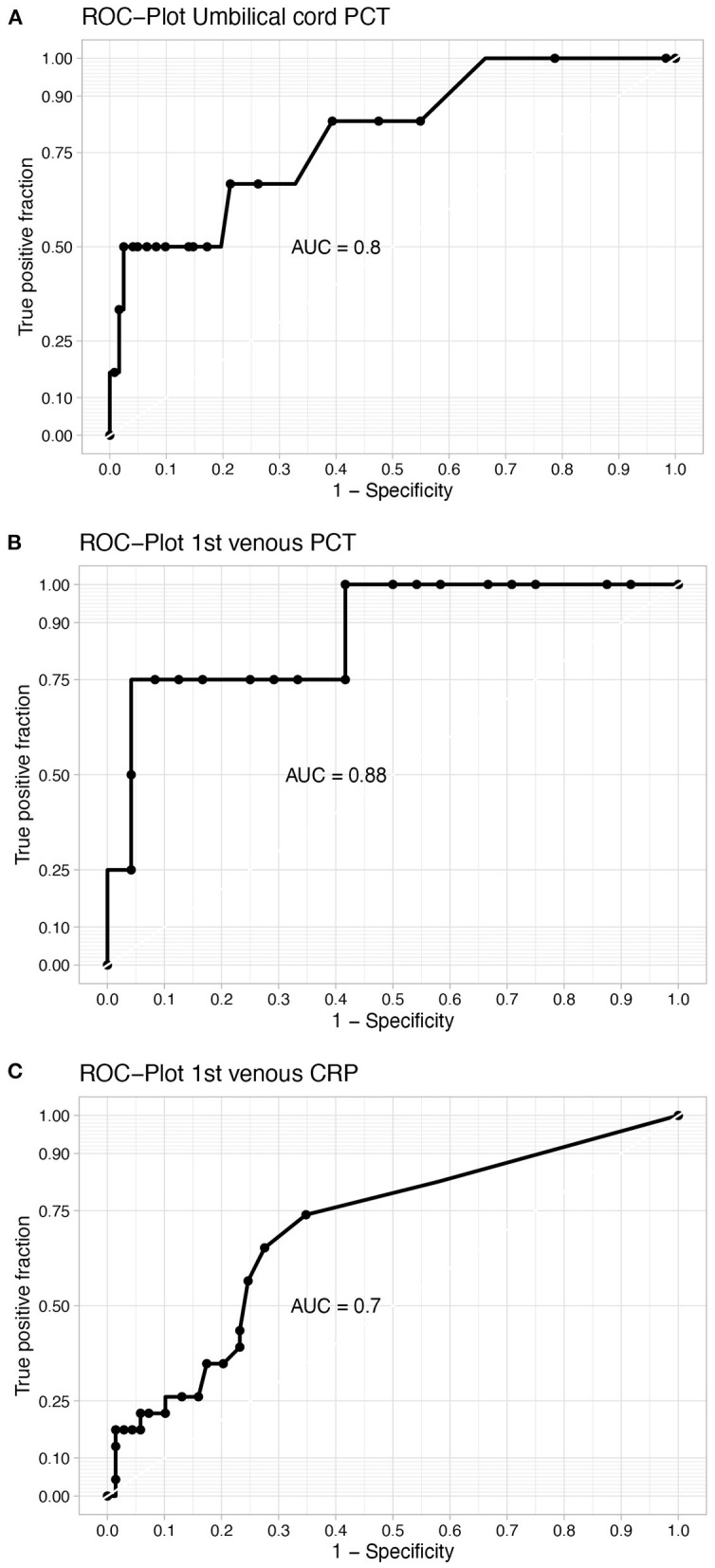
Sensitivity and specificity analysis. Receiver operating characteristic (ROC) curves of **(A)** umbilical cord PCT, **(B)** PCT in first venous blood sample postpartum, **(C)** CRP in first venous blood sample postpartum. AUC, Area under the curve.

In order to determine the diagnostic accuracy of PCT in both cord blood and blood samples postpartum, ROC curves were constructed and sensitivity/specificity analyses were performed. ROC curves show an AUC of 0.8 for PCT measured in umbilical cord blood (Figure 5). Youden index was used to determine the optimal cut-off value and showed a cord-blood PCT cut-off point of 2.03 ng/ml with a sensitivity and specificity of respectively 50% and 98%. Analysis of the effect of PCT cut-off levels on diagnostic accuracy using cross-tables showed a PCT cut-off point of 0.1ng/ml with a corresponding sensitivity of 83% and a specificity of 62% (Supplementary Table 2). When the cut-off level was increased to 0.6 ng/ml, based on previous research the sensitivity and specificity of umbilical PCT decreased to 50% and 95% respectively.

PCT directly postpartum has a sensitivity of 100% with a cut-off of 1.2 ng/ml and a specificity of 60%. The AUC was 0.875. CRP directly postpartum demonstrates a sensitivity of 82% with a specificity of 42% at the clinically not feasible cut-off of 0.5mg/L. When the threshold of CRP was increased to >10 mg/l to reflect current practice, this resulted in a sensitivity of 29% and a specificity of 86%.

## Discussion

This study examined the use of PCT as a biomarker to detect EOS in children born between 32 and 42 weeks. We have shown that newborns with a blood-culture confirmed or highly probable EOS (group A) present with a significantly increased umbilical cord blood PCT. Healthy controls and newborns who were not eligible for antibiotic therapy display a low PCT corresponding with their non-infectious state. Our focus was on newborns with an unclear clinical symptom presentation or more than one EOS risk factor (group B). Generally, these newborns are treated with antibiotics, although in practice, the antibiotics are often discontinued after 48-72 hours when their clinical condition improves, and the blood culture turns out negative. Treatment of these newborns contributes largely to neonatal antibiotic overtreatment. In this study, we show that group B presents with a low umbilical cord PCT, suggesting that antibiotic treatment might not have been necessary in the first place. However, a significant difference with group A could not be shown, most likely due to the small sample size of group A. The observed trend might suggest that PCT could be useful in distinguishing group B from truly infected newborns. This, however, will have to be evaluated in a larger cohort before it could be implemented in daily clinical practice. The value of PCT as an EOS diagnostic biomarker and follow-up metric to reduce antibiotic treatment duration has been shown before (20, 21). Our study confirms the diagnostic usability of PCT. It also quantifies the difference in PCT levels between newborns with probable EOS and newborns without EOS risk factors.

The ideal biomarker for EOS would be available at an early stage, not be affected by other factors, and can be obtained non-invasively. Umbilical cord sampling is a non-painful technique to obtain a sufficient amount of blood directly after birth. Another advantage of umbilical cord sampling is that it is performed at the start of the physiological peak of PCT, suggesting that an above-average PCT is caused by infection (21, 24, 25). Our study did not find a significant difference between PCT in arterial and venous cord blood, suggesting that both samples could be used. It should be noted that a significant difference between arterial and venous pH was also not found, raising the possibility that arterial and venous blood mixing occurred while obtaining the samples. If this was not the case, the measured PCT could represent an accurate measure of the PCT level in cord blood. Corresponding arterial and venous PCT values could also be explained by a combined intrinsic neonatal PCT production (leading to increased arterial values) and placental PCT production (resulting in increased venous values). PCT is produced by nearly all parenchymal tissues, most likely including the placenta. Alternative explanations for similar arterial and venous measurements include considerations of EOS associated infections and the physical size of the PCT molecule. EOS is related to intrauterine infection such as maternal chorioamnionitis; both conditions could result in elevated venous cord blood PCT. Additionally, the small molecular size of PCT (13 kDa) would allow for easy transfer of PCT through the placental capillary bed, leading to equal PCT values both in venous and arterial cord blood. Taken together, this suggests that both arterial and venous umbilical cord blood could be used, which improves the ease for using PCT for EOS detection.

Our data showed that antepartum maternal antibiotic administration was associated with lower PCT levels in both cord blood and blood samples directly postpartum. Hypothetically, maternal antibiotics are effective in controlling maternal and/or intra-uterine infection resulting in a lower immune response and PCT level. The association between maternal antibiotics and lower PCT levels could not be shown for newborns with proven or probable EOS, due to the small sample size, but this would be essential to prove the abovementioned hypothesis. A larger study cohort could clarify if PCT is indeed a marker for effective maternal therapy. An alternative explanation might be a (temporarily) suppression of PCT production by antibiotic treatment. This would indicate that PCT alone cannot guide therapeutic decision-making in newborns of mothers who received antibiotics. However, to our knowledge, no direct pharmacodynamic process of antibiotics has been described that could alter PCT levels.

PCT cut-off points vary widely in literature ranging between 0.004 ng/ml and 6 ng/ml. Previous studies using cut-off values comparable to our study report a PCT sensitivity similar to our findings in postpartum neonatal blood (16, 26, 27). Currently, techniques can detect low PCT levels and allow for dealing with cut-offs such as 0.1 ng/ml. In our study, a PCT cut-off value of 0.1 ng/ml corresponds with a sensitivity of 83%. This is in line with the available cord blood PCT reference interval data (28). Increasing the cut-off value to 0.6 ng/ml, such as in Huetz et al., would lower the sensitivity and specificity of umbilical PCT in our dataset to respectively 50% and 93%, which is undesirable (22). We, therefore, recommend a PCT threshold of 0.1 ng/ml to guide antibiotic decision making, as suggested by our data.

Limitations of our study include the limited availability of useable samples, the monocentric design, and the low incidence of proven EOS. As pH analysis in the cord blood samples and CRP measurements in postpartum blood samples were done first, minimal volumes of remaining blood would be available for measuring PCT levels. This resulted in a small group of cases with useful PCT samples (group A) and subsequent unequal group sizes. These group sizes complicated statistical analysis and possibly resulted in a smaller effect than we could identify in the current set-up. The low number of EOS cases is a limitation that several multicenter EOS studies have faced (3). Our study also had several culture-negative EOS cases. This could be addressed by gathering data on an (inter)national level in the future. Lastly, we were confronted with a change in the local antibiotic treatment protocol due to new scientific insights during this study. Both antibiotic combinations were at the time in agreement with the Dutch sepsis protocol. As this study focuses on pre-treatment PCT values, we do not expect this to affect our results.

As many studies have shown before, the ideal independent biomarker has not been identified to date. The sensitivity and specificity in our study confirm that PCT alone is not the answer. A promising approach to improve EOS diagnosis is a screening tool with one or more biomarkers (29). The EOS calculator proposed by Escobar is an easy to use interactive tool for predicting the probability of EOS (11, 30). This calculator combines both maternal intrapartum risk factors as well as neonatal clinical risk factors, and has been shown to be equal to the current guidelines in terms of safety (14, 31). A recent retrospective analysis showed a fourfold reduction in antibiotic use when decision-making was based on the EOS calculator rather than the current Dutch guidelines (13). However, the EOS calculator has been shown to perform poorly in identifying EOS at the early stage of infection and in predicting which newborns will develop sepsis in the coming hours (14, 15). A strategy to improve diagnostic accuracy is combining a risk factor-based approach, like the EOS calculator, with umbilical cord blood PCT. This combination has been shown to substantially reduce antibiotic overtreatment without increasing the number of missed infections (22). Furthermore, bedside PCT would improve the time to diagnosis and can accurately detect bacterial infection in young infants (32, 33). Therefore, the combination of the EOS calculator, a bedside PCT, and potentially additional biomarkers promises to improve EOS detection even further.

In conclusion, umbilical cord blood PCT levels are increased in newborns ≥32 weeks with a proven or probable EOS and low in newborns with only risk factors for infection. This suggests that PCT could be useful to distinguish infected from non-infected newborns with or without EOS risk factors.

## Data Availability Statement

The original contributions presented in the study are included in the article/supplementary material, further inquiries can be directed to the corresponding author.

## Ethics Statement

The studies involving human participants were reviewed and approved by the Medical Ethical Committee of the Amsterdam UMC on March 18th, 2019 (ID: NL67266.029.18). Written informed consent to participate in this study was provided by the participants' legal guardian/next of kin.

## Author Contributions

OD, LL, and MH designed and performed the study and wrote the paper with input from all authors. Data collection was performed by OD, with help of LL, PG, KV, and IS. BW performed sample processing and biomarker tests. OD, LL, and SE performed the data analyses. MH supervised the project.

## Funding

The research was supported by a research fund of the Spaarne Gasthuis Academy.

## Conflict of Interest

The authors declare that the research was conducted in the absence of any commercial or financial relationships that could be construed as a potential conflict of interest.

## Publisher's Note

All claims expressed in this article are solely those of the authors and do not necessarily represent those of their affiliated organizations, or those of the publisher, the editors and the reviewers. Any product that may be evaluated in this article, or claim that may be made by its manufacturer, is not guaranteed or endorsed by the publisher.
