# Supplementary material for: Umbilical Cord Procalcitonin to Detect Early-Onset Sepsis in Newborns: A Promising Biomarker
Source: Front Pediatr. 2021 Dec 10;9:779663. doi: 10.3389/fped.2021.779663 (PMC8704118; doi:10.3389/fped.2021.779663)
Supplement: Supplementary file 1 [file Data_Sheet_1.docx]

**Supplemental material**

**Supplemental table S1. Maternal and neonatal EOS risk factors**

Neonatal early-onset sepsis risk factors as defined by the Paediatric Association of the Netherlands (NVK) 2017 EOS guideline. The guideline is an adaptation of the U.K. NICE (National Institute for Health and Care Excellence) guideline. Based on the NVK recommendations, antibiotic treatment should be started when 2 or more risk factors are present.

|  |  |
| --- | --- |
| Maternal risk factors for EOS – NVK 2017 |  |
| 1. Parenteral antibiotic treatment given to the mother for confirmed or suspected invasive bacterial infection at any time during labour, or in the 24-hour periods before and after the birth. | 1. Prelabour rupture of membranes >24 hours in a term pregnancy |
| 1. Suspected or confirmed infection in another baby in the case of a multiple pregnancy. | 1. Spontaneous preterm birth (gestational age <37 weeks) |
| 1. Invasive group B streptococcal infection in a previous baby. | 1. Suspected or confirmed rupture of membranes for more than 18 hours in a preterm birth |
| 1. Maternal group B streptococcal colonisation, bacteriuria or infection in the current pregnancy | 1. Intrapartum fever higher than 38°C, or confirmed or suspected chorioamnionitis |

|  |  |
| --- | --- |
| Neonatal risk factors for EOS – NVK 2017 |  |
| 1. Respiratory distress >4 hours postpartum | 1. Hypoxia (e.g. central cyanosis or low O2 sat.) |
| 1. Neonatal epileptic seizures | 1. Neonatal Encephalopathy |
| 1. Intubation of a term born neonate | 1. The necessity for cardiopulmonary resuscitation |
| 1. Signs of shock | 1. Mechanical ventilation in a premature neonate |
| 1. Behavioural change (silent neonate, hypotonia) | 1. Persistent pulmonary hypertension |
| 1. Trouble with feeding | 1. Fever(>38ºC) of hypothermia (<36°C) |
| 1. Apnoea and bradycardia | 1. Local signs of infection (e.g. on the skin or eyes) |
| 1. Signs of respiratory distress |  |

**Supplemental table S2. Sensitivity and specificity analysis**

Cross-tabulation of PCT and CRP measurements with corresponding sensitivity, specificity, positive predictive value and negative predictive values. Group B and C are taken as control groups: those neonates were only at risk for EOS but were not treated as such with >72 hours antibiotics due to good clinic and a negative blood culture.

|  | Positive | Negative |  | Predictive values |
| --- | --- | --- | --- | --- |
| umbilical PCT cut-off 0.1 ng/ml | **Group A** – cases, n | **Group B and C –** controls, n | **Total** |  |
| PCT> 0.1 ng/ml | 5 | 54 | 59 | Positive predictive value – 9.4% |
| PCT< 0.1 ng/ml | 1 | 64 | 65 | Negative predictive value – 98.7% |
| Total | 6 | 118 | 124 | - |
| Sensitivity and specificity | Sensitivity: 83.3% | Specificity 61.8% | - | - |

|  | Positive | Negative |  | Predictive values |
| --- | --- | --- | --- | --- |
| umbilical PCT cut-off 0.6 ng/ml | **Group A** – cases, n | **Group B and C –** controls, n | **Total** |  |
| PCT> 0.6 ng/ml | 3 | 6 | 9 | Positive predictive value – 33.3% |
| PCT< 0.6 ng/ml | 3 | 116 | 119 | Negative predictive value – 97.5% |
| Total | 6 | 122 | 128 | - |
| Sensitivity and specificity | Sensitivity: 50.0% | Specificity 95.1% | - | - |

|  | Positive | Negative |  | Predictive values |
| --- | --- | --- | --- | --- |
| umbilical PCT cut-off 2.03 ng/ml | **Group A** – cases, n | **Group B and C –** controls, n | **Total** |  |
| PCT> 2.03 ng/ml | 3 | 3 | 6 | Positive predictive value – 50% |
| PCT< 2.03 ng/ml | 3 | 119 | 122 | Negative predictive value – 97.5% |
| Total | 6 | 122 | 128 | - |
| Sensitivity and specificity | Sensitivity: 50.0% | Specificity 97.5% | - | - |

|  | Positive | Negative |  | Predictive values |
| --- | --- | --- | --- | --- |
| venous PCT cut-off 1.2 ng/ml | **Group A** – cases, n | **Group B and C –** controls, n | **Total** |  |
| PCT> 1.2 ng/ml | 4 | 10 | 14 | Positive predictive value – 28.6% |
| PCT< 1.2 ng/ml | 0 | 15 | 15 | Negative predictive value – 100% |
| Total | 4 | 25 | 29 | - |
| Sensitivity and specificity | Sensitivity: 100.0% | Specificity 60.0% | - | - |

|  | Positive | Negative |  | Predictive values |
| --- | --- | --- | --- | --- |
| venous CRP cut-off 10 ng/ml | **Group A** – cases, n | **Group B and C –** controls, n | **Total** |  |
| CRP> 10 mg/l | 7 | 10 | 17 | Positive predictive value – 41.2% |
| CRP< 10 mg/l | 17 | 59 | 76 | Negative predictive value – 77.6% |
| Total | 24 | 69 | 93 | - |
| Sensitivity and specificity | Sensitivity: 29.2% | Specificity 85.5% | - | - |
